# Two Cases of Pigmented Basal Cell Carcinoma in African American Patients

**DOI:** 10.7759/cureus.62862

**Published:** 2024-06-21

**Authors:** Angelique Ruml, Joan K Fernandez, Ibeth Caceres, Nisha Ramani, Ida Orengo, Theodore Rosen

**Affiliations:** 1 Dermatology, Baylor College of Medicine, Houston, USA; 2 Dermatology, Michael E. DeBakey Veterans Affairs Medical Center, Houston, USA; 3 Pathology and Immunology, Baylor College of Medicine, Houston, USA; 4 Pathology and Immunology, Michael E. DeBakey Veterans Affairs Medical Center, Houston, USA

**Keywords:** nonmelanoma skin cancer, skin of color, nevus sebaceous, basal cell carcinoma, pigmented basal cell carcinoma, case report

## Abstract

Basal cell carcinoma (BCC) is the most common cutaneous malignancy, comprising approximately 80% of non-melanoma skin cancers. There are numerous subtypes, including pigmented basal cell carcinoma (pBCC), a rare clinical and histological variant. Skin cancers in African American patients, although rare, still do occur. BCC is an uncommon neoplasm in this population, but when it does occur, pigmentation is present in more than 50% of tumors compared with only 5% to 6% of BCCs in Caucasians. This report presents two cases of histologically verified pBCC in African American patients from dermatology clinics at the Veterans Affairs Hospital located in the Texas Medical Center. With the population of the United States growing more diverse, these cases emphasize the importance of recognizing the nuanced morphology of BCC in the skin of color compared to lighter-skinned counterparts. This is especially necessary, as early detection and prompt management are key to combating the disproportionately high morbidity and mortality related to skin cancers affecting patients of color.

## Introduction

Current estimates are that one in five Americans will develop skin cancer in their lifetime, and cases of non-melanoma skin cancers are rising [1]. Basal cell carcinoma (BCC) was first described in the year 1827 by Jacob, and it is the most common type of skin cancer [2]. It classically appears as a slow-growing, translucent elevated lesion on sun-exposed areas, most commonly located in the head and neck region [3]. Risk factors include an extensive history of sun exposure (particularly those with Fitzpatrick skin phototypes I-III), as well as exposure to arsenic, coal tar derivatives, and irradiation.

Annually, it is estimated that approximately four million Americans are diagnosed with BCC, exceeding the incidence of all other cancers combined [4,5]. There are numerous subtypes of BCC, including pigmented basal cell carcinoma (pBCC), which comprises approximately 6% of all diagnosed BCCs [3]. These pathologic subtypes are characterized by the accumulation of melanin, melanocytes, and melanophages in the tumor nodules, giving rise to the pigmentation seen clinically. Irrespective of the pathologic subtype, wide local excision and Mohs micrographic surgery (MMS) remain first-line treatments. The appropriate use criteria for MMS may be used to identify instances in which MMS is recommended over wide local excision. Generally, MMS is favored for aggressive BCC subtypes (i.e., morpheaform, infiltrating, micronodular, etc.), recurrent BCC of any size, an unexpected positive margin on a recent excision, and lesions in cosmetically sensitive areas [6].

The incidence of cutaneous malignancies remains relatively low in darker-skinned patients due to enhanced protection due to increased melanin production. The incidence of skin cancer among non-Hispanic White individuals is almost 30 times higher than that among non-Hispanic Black or Asian/Pacific Islander individuals [4]. However, morbidity and mortality are disproportionately higher in minority groups, as these malignancies are often diagnosed in later stages, where they may become more difficult to treat and tend to behave more aggressively [7,8].

This presents a diagnostic challenge for those caring for patients with skin of color. Classically, BCCs present as pearly papules or nodules with rolled borders and overlying telangiectasias, elements that may be difficult to visualize in darker skin tones. BCCs in black patients are almost always pigmented, a finding that may lead to the consideration of alternate diagnoses, including malignant melanoma, melanocytic nevus, and pigmented seborrheic keratosis, to name a few [9]. Dermoscopy may be a useful tool in diagnosis; features such as maple-leaf areas with peripheral projection, spoke wheel areas, blue-gray dots, superficial fine telangiectasias, small erosions, and shiny white-red structureless areas may be observed in pBCC [10].

Here, we present two cases of pBCC in African American patients. Although similar cases have previously been described in the literature, given the continued increased morbidity and mortality due to significantly delayed diagnosis in this patient population, it is evident that we need to continue to call attention to such cases. This report will detail our patients’ clinical presentations, including the morphologic and pathologic characteristics of their neoplasms.

## Case presentation

**Figure 1:**
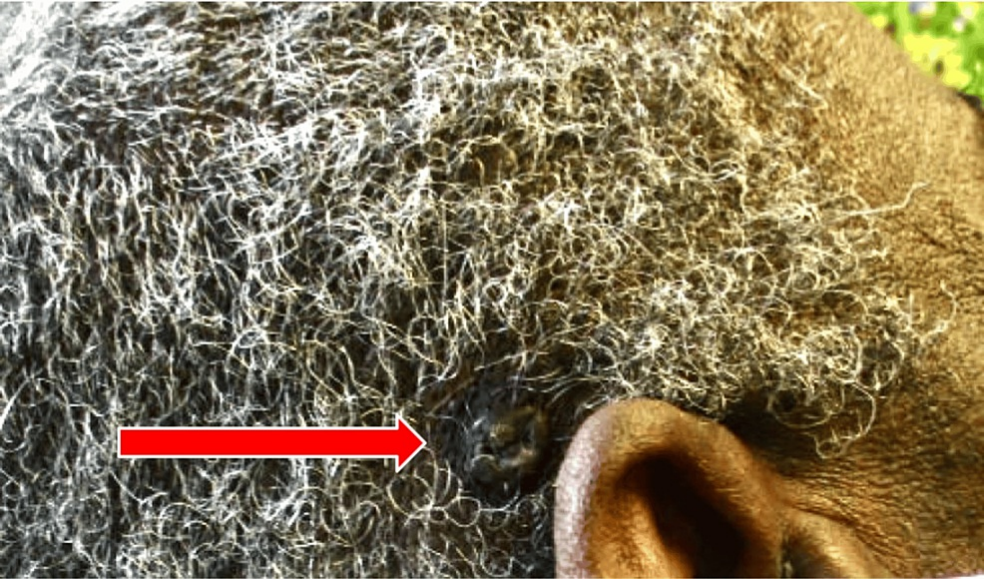
1.3 x 1.3 cm hyperpigmented plaque with central punctum noted on the right temporal scalp

**Figure 2:**
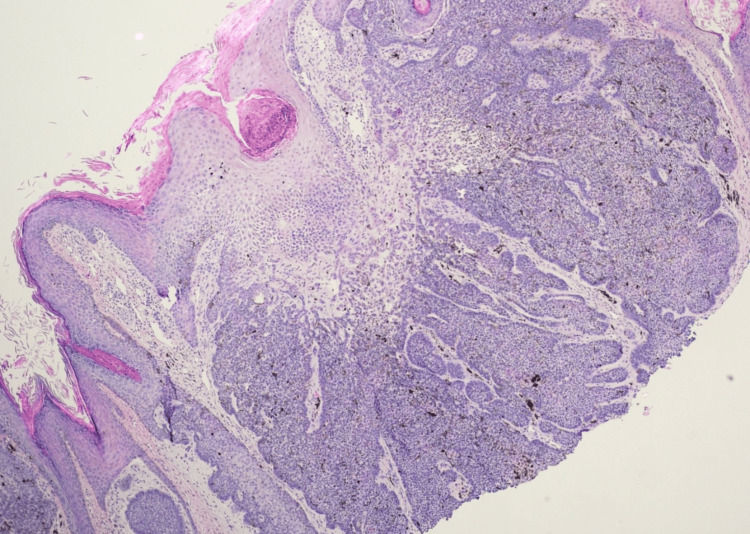
Shave biopsy reveals a well-circumscribed nodule consisting of basaloid nests with mucin production and tumoral and stromal melanophages

**Figure 3:**
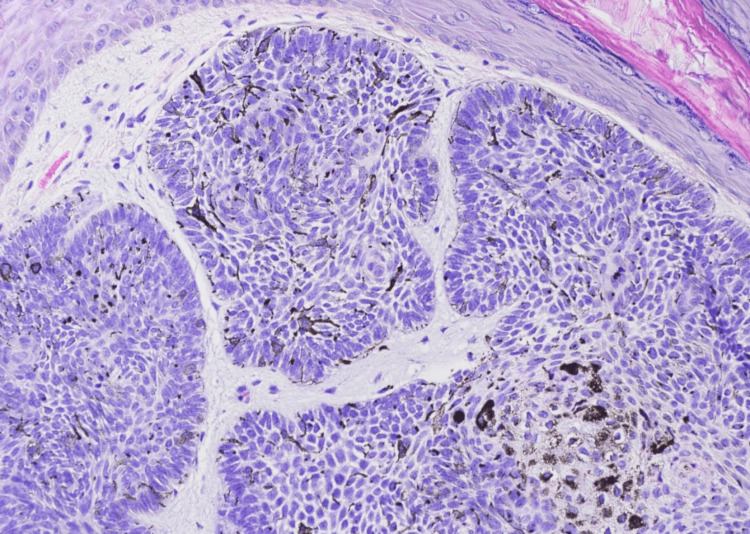
Section shows a solid lobule of basaloid cells with peripheral nuclear palisading and scattered melanophages

Our first patient is a 75-year-old black male with a past medical history significant for treated late latent syphilis and HIV well controlled on HAART (elvitegravir, cobicistat, emtricitabine, and tenofovir alafenamide) and no history of skin cancer who presented with a painless nodule on the right temporal scalp for the last two years. The lesion had drained liquid in the past and was newly firm. On examination, the patient was found to have a firm, hyperpigmented plaque with a central punctum measuring 1.3 x 1.3 cm (Figure 1). The differential diagnosis included a pilar cyst with surrounding scar tissue versus seborrheic keratosis versus folliculitis. The patient opted for monitoring at that time. He returned to the clinic two months later for re-evaluation, given worsening pain, scaling, and itching. At that time, a shave biopsy of the lesion was performed, and pathology confirmed the diagnosis of a pigmented basal cell carcinoma with superficial and nodular patterns extending to the base of the biopsy (Figures 2, 3). The patient was treated with MMS, and negative margins were achieved in one stage. The defect was closed with an intermediate layered repair, and the patient tolerated the procedure well without complications.

**Figure 4:**
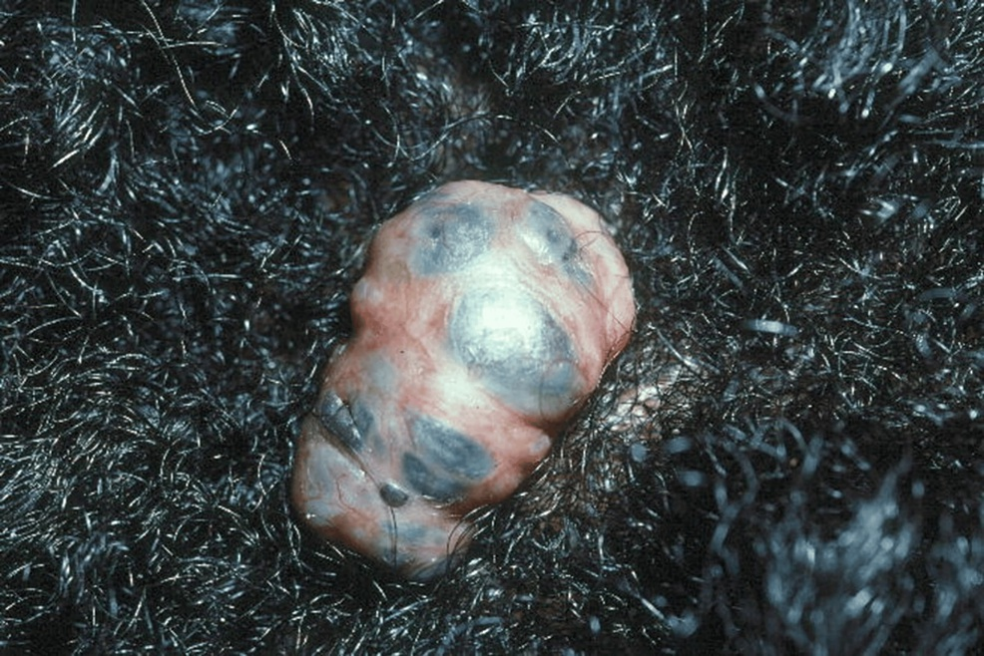
2.5 x 1.5 cm pedunculated nodule with areas of macular pigmentation on the scalp

**Figure 5:**
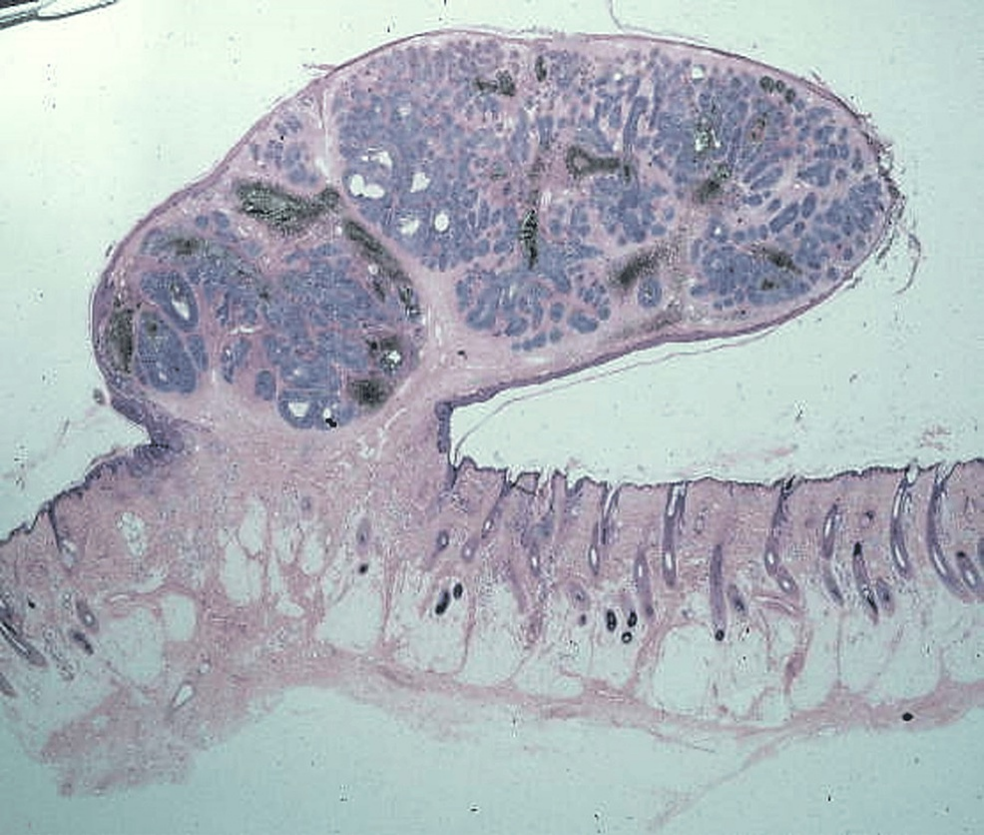
Section shows basaloid nests with mucin production and scattered tumoral and stromal melanophages

Our second patient is a 27-year-old black male with no significant past medical history who presented with a painless nodule on his scalp that he noticed approximately three months before presentation. The patient reported that the lesion was asymptomatic but had gradually become more raised over time, prompting his barber to bring it to his attention and recommend an evaluation. On physical exam, a 2.5 x 1.5 cm pedunculated nodule with areas of macular pigmentation was noted on the scalp (Figure 4). A shave biopsy was performed, and pathology revealed a pigmented basal cell carcinoma arising within a nevus sebaceous (Figure 5). The patient was treated with wide local excision with negative margins and had no evidence of recurrence at his 10-year follow-up.

## Discussion

Many factors contribute to the development of non-melanoma skin cancers, including both genetic and environmental factors. It is well-established that exposure to ultraviolet radiation (UVR) leads to DNA mutations within the nuclei of epidermal keratinocytes, oxidative stress, and inflammatory responses - all of which play a pivotal role in skin cancer genesis [11].

The incidence of skin cancers in patients with Fitzpatrick skin phototypes V & VI is lower compared to their fairer-skinned counterparts. Patients with darker skin have increased epidermal melanin, namely increased eumelanin, compared to patients with lighter skin. Melanin, especially eumelanin, possesses a shielding effect against UVR by not only serving as a physical barrier that scatters UVR but also by acting as an absorbent filter that reduces the penetration of UVR through the epidermis [12]. The increased protective effects of melanin possessed by patients with darker skin types result in a decreased overall incidence of skin cancer in this patient population.

While patients with darker skin have extra protection against the harmful effects of UVR, they do continue to run the risk of developing cutaneous carcinoma. While squamous cell carcinoma (SCC) is the most frequent neoplasm encountered, BCC is also seen, contributing to 20%-30% of skin cancers in people of color [11]. It is well-documented that pigmented BCC is more common in patients with darker skin. Altman et al. remarked that BCCs in black patients are almost always pigmented, a finding that can lead to erroneous diagnoses such as malignant melanoma, particularly invasive nodular melanoma, melanocytic matricoma, or pigmented seborrheic keratosis [9]. Because of this, we cannot rely on the classic textbook morphology of BCC, more commonly noted in lighter-skinned patients: pink pearly papules with rolled borders, central ulceration, and telangiectasias. It is important to note that telangiectasias that classically occur in conjunction with BCC are more difficult to detect in our darker-skinned patients or lesions with background pigmentation. 

Because the classically taught morphology of BCCs may not be present in darker-skinned patients, it is important to have a high index of suspicion in patients presenting with new or changing lesions, especially those with associated symptoms: tenderness, ulceration, friability, or pruritus. The second patient case presented above is unique in that not only is the morphology nonclassical for BCC but also the lesion developed in a preexisting congenital nevus sebaceous. Nevus sebaceous are congenital hamartomas of the pilosebaceous follicular unit, with the vast majority occurring on the head and neck. These growths commonly present at birth or develop in early childhood, but in adulthood, the growths may develop secondary neoplasms within them. There have been many reports of the development of both benign and malignant growths within them, most commonly trichoblastoma (or melanotrichoblastoma, its pigmented variant that can be seen in individuals of color), but BCC has also been found [9,12-14]. The process of malignant degeneration is often accompanied by pigmented color change, rapid change in size, ulceration, pruritus, pain, bleeding, or ulceration [15]. Ulceration can also be seen with SCC, which tends to appear as a sore that will not heal (with bleeding or crusting) as opposed to the black, pearly appearance more characteristic of BCC seen in patients of color [11,16]. Clinicians should be suspicious of malignant transformation for any lesion with rapid morphological changes and have a low threshold for biopsy to rule out possible underlying neoplasia [13].

The treatment of BCC in black patients is the same as in other patients [9]. The gold standard treatment for pBCC is based upon both the size and location of the lesion. Electrodesiccation and curettage, wide excision, and MMS are all appropriate therapeutic options [9]. BCC is typically curable when the diagnosis is made promptly, and the lesion is treated in a timely manner. Because of its high incidence, BCC constitutes an enormous financial burden on the health care system [2]. Thus, increased awareness of this condition can help to aid accurate diagnosis and prompt management. Delay to diagnosis is particularly important to be aware of in patients with darker skin types, as disparities in access to dermatologic care disproportionately affect minority populations [17].

## Conclusions

These cases underscore the rarity and diagnostic challenges posed by pBCC in patients with darker skin. The lesions may not appear as they classically do in those with fairer skin, so scrutiny is warranted on skin examination, as these tumors may be indistinguishable from background pigmentation. These cases highlight the importance of keeping pBCC on the differential diagnosis in patients presenting with a suspicious pigmented lesion, especially on the head and neck. This clinical vigilance will facilitate prompt diagnosis and treatment, subsequently helping to improve patient outcomes.
